# Efficacy and Safety of Cone-Beam CT Augmented Electromagnetic Navigation Guided Bronchoscopic Biopsies of Indeterminate Pulmonary Nodules

**DOI:** 10.3390/tomography8040172

**Published:** 2022-08-18

**Authors:** Shreya Podder, Sana Chaudry, Harpreet Singh, Elise M. Jondall, Jonathan S. Kurman, Bryan S. Benn

**Affiliations:** Department of Pulmonary and Critical Care Medicine, Medical College of Wisconsin, 8701 W Watertown Plank, Milwaukee, WI 53226, USA

**Keywords:** electromagnetic navigation bronchoscopy, cone beam computed tomography, pulmonary nodule, cancer

## Abstract

Bronchoscopic biopsy results for indeterminate pulmonary nodules remain suboptimal. Electromagnetic navigation bronchoscopy (ENB) coupled with cone beam computed tomography (CBCT) for confirmation has the potential to improve diagnostic yield. We present our experience using this multimodal approach to biopsy 17 indeterminate nodules in 14 consecutive patients from April to August 2021. Demographic information, nodule characteristics, and biopsy results were recorded. Procedures were performed in a hybrid operating room equipped with a Siemens Artis Q bi-plane CBCT (Siemens, Munich, Germany). After ENB using the superDimension version 7.1 (Medtronic, Plymouth, MN, USA) to target the lesion, radial endobronchial ultrasound was used as secondary confirmation. Next, transbronchial needle aspiration was performed prior to CBCT to evaluate placement of the biopsy tool in the lesion. The average nodule size was 21.7+/−15 mm with 59% (10/17) < 2 cm in all dimensions and 35% (6/17) showing a radiographic bronchus sign. The diagnostic yield of CBCT-guided ENB was 76% (13/17). No immediate periprocedural or postprocedural complications were identified. Our experience with CBCT-guided ENB further supports the comparable efficacy and safety of this procedure compared to other mature biopsy modalities. Studies designed to optimize the lung nodule biopsy process and to determine the contributions from different procedural aspects are warranted.

## 1. Introduction

Data from multiple randomized controlled clinical trials have shown the benefit of low dose computed tomography (CT) scans on lung cancer detection and in reducing mortality [1,2]. The recently updated lung cancer screening guidelines are expected to expand the number of eligible patients for this imaging study [3]. Similarly, the number of incidentally found pulmonary nodules is also increasing, with an incidence that is much greater than previously estimated [4]. Although most identified nodules are benign and do not require further evaluation [5], a portion of those discovered will need serial imaging or tissue diagnosis prior to treatment. 

Assessing indeterminate or intermediate risk pulmonary nodules is especially difficult. While physicians rely on established risk calculators [6,7] or clinical acumen [8], these approaches are fraught with inaccuracies and inconsistencies [9]. When tissue diagnosis is required, guidelines recommend transthoracic needle aspiration (TTNA) biopsy, bronchoscopic biopsy, or surgery [10]. Although TTNA may be useful in obtaining tissue samples from peripheral nodules with high accuracy [11], there is a significant risk of pneumothorax requiring chest tube drainage and bleeding, especially when sampling more central lesions [11].

Bronchoscopy has evolved over the years, allowing practitioners to accurately sample endobronchial and larger parenchymal lesions [12]. However, small (<2 cm), peripheral, lesions that lack a radiographic bronchus sign (an airway immediately adjacent to, or directly aligned with, the lesion) still pose a significant challenge [13,14]. Electromagnetic navigation guided bronchoscopy (ENB) has increased the reach of bronchoscopic procedures [12], but the sensitivity for malignancy is still suboptimal [15]. Although guided bronchoscopy does have improved yields compared to non-guided procedures [14], the overall results fail to rival that of TTNA. 

Factors that affect the diagnostic yield of ENB guided biopsy are CT scan to body divergence (CTBD) and the absence of continuous imaging during tissue sampling. CTBD refers to the difference in target lesion location on the preprocedural CT scan used for virtual mapping of the pathway to the lesion compared to the actual lesion location in the patient’s lung parenchyma during the procedure. CTBD may lead to ~18 mm difference in expected and actual nodule location due to respiratory motion [16]. Efforts to overcome CTBD include anesthesia protocols and ventilation strategies optimized to reduce motion and minimize atelectasis [17]. Fluoroscopic navigation with digital tomosynthesis allows for an updated nodule location prior to biopsy to decrease CTBD and has been shown to improve diagnostic yield compared to traditional ENB [18]. The addition of real-time guidance catheter tip tracking also increases diagnostic yield [19].

Another promising option to improve the diagnostic yield of lung nodule biopsies is to utilize cone beam CT (CBCT). This approach seeks to overcome CTBD by locating the pulmonary lesion in real time and allows for real-time assessment of the interaction between biopsy tools and lesion [20]. We recently implemented this process at our institution and present our initial results for diagnostic yield when coupling ENB with CBCT. 

## 2. Methods

### 2.1. Study Cohort

Fourteen patients presenting to our interventional pulmonology clinic for evaluation of lung nodules from April through August 2021 were consented for CBCT-guided ENB and enrolled. Demographic data, nodule characteristics, biopsy results, and complications from the procedure, including pneumothorax and bleeding severity, were recorded. Benign histological findings that support a nodule diagnosis were classified according to previously published recommendations [19]. The study was approved by the institutional review board of the Medical College of Wisconsin PRO00036023. 

### 2.2. Procedure Description

Prior to the procedure, the patient’s most recent CT chest was uploaded into the superDimension version 7.1 (Medtronic, Plymouth, Minnesota) ENB system for preprocedural planning in which the target lesion is identified to create a virtual bronchoscopy map for guidance during the ENB portion of the procedure. All procedures were performed in a hybrid operating room equipped with a Siemens Artis Q bi-plane CBCT (Siemens, Germany). General anesthesia with neuromuscular blockade, fraction of inspired air between 40−60%, a tidal volume of 6–8 cc/kg ideal body weight to mirror physiologic lung volumes, and positive end expiratory pressure of at least 5 cm H_2_O was used. A flexible bronchoscope (Olympus BF-1TH190, inner diameter 2.8 mm Olympus America, field of view 120°, direction of view 0° forward viewing, depth of field 3–100 mm, Center Valley, PA, USA) was used for airway inspection through an 8.5 mm endotracheal tube. 

After completion of airway inspection, the ENB portion of the procedure was performed. Catheter angle was selected at the discretion of the operator. The extended working channel (EWC) and locatable guide (LG) were coupled together and inserted into the working channel for automatic registration, which is performed to align the patient’s central airway with the virtual bronchoscopy images for appropriate alignment to decrease the risk of CTBD. Peripheral navigation to the target lesion was then performed with guidance from the ENB system.

**Figure 1:**
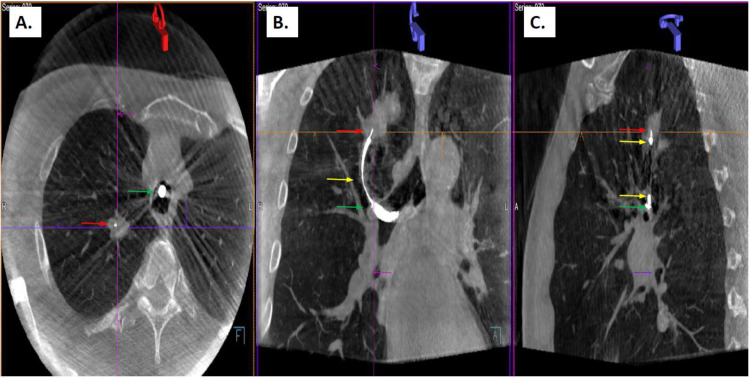
Representative Cone Beam Computed Tomography imaging showing interaction between biopsy tool (red arrow head, 21-gauge Arcpoint^TM^ needle (Medtronic, Plymouth, Minnesota)) placed through extended working channel (yellow arrow head) of electromagnetic navigation system (superDimension version 7.1 (Medtronic, Plymouth, Minnesota)) inside the flexible bronchoscope (green arrow head) and the target peripheral pulmonary nodule in the right upper lobe (**A**) axial reconstruction, (**B**) coronal reconstruction, (**C**) sagittal reconstruction) (purple arrows and lines are part of the Siemens software analysis program).

After completion of local registration, the LG was removed, and a radial endobronchial ultrasound (REBUS) probe was inserted through the EWC. A 21-gauge Arcpoint^TM^ needle (Medtronic, Plymouth, MN, USA) was then inserted into the target lesion and an initial CBCT was performed for secondary confirmation, defined as visualization of the needle inside the lesion in three imaging planes (anterior-posterior, 25 degrees left anterior oblique, 25 degrees right anterior oblique) (Figure 1). CBCT was performed while the patient was apneic to mitigate the risk of respiratory motion degrading the quality of the obtained images. Each CBCT spin was performed with an imaging acquisition time of six seconds. Images were then reconstructed for review at a separate workstation. Additional catheter manipulations, if needed, were guided by the initial CBCT results. Additional CBCT spins were performed at the discretion of the proceduralist. Biopsies were then performed with fluoroscopic guidance. After procedure completion, the EWC was removed and bronchoscopic visualization of the airway was performed to assess for bleeding. Once airway hemostasis was confirmed, a post-procedure chest x-ray was obtained to evaluate for pneumothorax. 

### 2.3. Statistical Analyses

All statistical analyses were performed using Microsoft Excel (Microsoft Corporation, Redmond, WA, USA). The primary endpoint was diagnostic yield, which was defined as the total number of nodules with a diagnostic biopsy (including malignancy or benign findings such as inflammation or infection) divided by the total number of nodules biopsied. The view on radial endobronchial ultrasound was evaluated after ENB was performed, but prior to the initial placement of biopsy tool and initial CBCT spin. The biopsy tool-lesion relationship refers to findings after the initial CBCT spin and was categorized as within (biopsy tool visualized inside of lesion), adjacent (biopsy tool visualized next to lesion, but not inside) or absent (no evidence of biopsy tool inside or next to lesion). Secondary endpoints included any procedural complications, including pneumothorax or bleeding. Effective radiation dose was calculated by summing total fluoroscopy time, including all CBCT spins and fluoroscopic guided biopsies performed during the procedure, and converting mGy to mSv. 

## 3. Results

In total, 17 pulmonary nodules were biopsied in 14 consecutive patients. Average patient age was 71 (62–84) years, and 43% (six in 14) were female (Table 1). Furthermore, 64% (nine in 14) were former or current smokers, and 43% (six in 14) had a history of cancer. 

The right upper lobe was the most common lesion site 47% (eight in 17) and the average nodule size was 21.7+/−15 mm (axial) x 15+/−9 mm (coronal) with 35% (six in 17) showing a radiographic bronchus sign (Table 2). REBUS revealed a concentric view in 24% (four in 17) prior to CBCT. After initial CBCT spin, there was evidence of the biopsy tool within 41% (seven in 17) of the targeted lesions. The average number of CBCT spins was 3.5+/−1.5 and the effective dose was 858.5+/−553 mGy. 

Diagnostic yield from the CBCT-guided ENB procedure was 76% (13/17), with 59% (10 in 17) malignant, 12% (two in 17) infectious, and 6% (one in 17) inflammatory (multinucleated giant cells and abundant histiocytes were seen with interval improvement on follow up imaging) (Table 3). Benign nodules were followed on serial imaging for at least eight months post procedure. Overall, 67% (two in three) of the benign nodules showed decreased size or resolution on subsequent CT scan. No periprocedural or immediate post procedural complications were identified. 

## 4. Discussion

While the technology for bronchoscopic lung nodule biopsies continues to improve, inherent challenges of the procedure lead to poor sensitivity. Novel approaches to overcome the effects of a dynamic, moving lung and a static, fixed preprocedural image exist. We leveraged CBCT guidance to biopsy 17 indeterminate pulmonary nodules with a favorable safety profile and comparable results to other mature, guided bronchoscopy strategies. 

The use of a preprocedural chest CT scan to create a virtual map to the target pulmonary nodule is a requirement for all ENB procedures that presents challenges. For myriad reasons, including insurance authorization, patient availability, and patient concern for additional radiation exposure, chest CT scans are often obtained weeks prior to the procedure rather than on the same day [21]. CBCT allows for real-time, intraprocedural assessment of pulmonary nodules. If a target lesion shows interval decrease in size or resolution, then biopsy may no longer be warranted. This information has the potential to decrease unnecessary procedures, as seen when performing a same day preprocedural chest CT prior to guided bronchoscopic biopsy [21]. Further studies to determine the optimal time of the initial CBCT spin, e.g., prior to the induction of general anesthesia versus after the biopsy tool has been used to access the lesion, is an area for future evaluation.

Because thorough airway visualization contributes significantly to the accuracy of the airway mapping, chest CT scans are ideally acquired at total lung capacity in a cooperative patient [22] with arms raised over their head [20]. ENB, however, is performed under moderate sedation or general anesthesia with arms tucked at the side. As a result, patients are often breathing at volumes approaching functional residual capacity or below tidal volume if there is significant postintubation atelectasis [23]. These respiratory parameters create a mismatch between the virtual map based on the chest CT and the patient’s actual anatomy, leading to CTBD, which varies between 6 to 30 mm in the upper lobes and 6 to 60 mm in lower lobes during a respiratory cycle [16]. This respiratory variation can impact the diagnostic yield. During our procedure, we employed a standardized anesthesia protocol using a tidal volume of 6–8 mL/kg of ideal body weight, at least 5 cm H_2_O positive end expiratory pressure, and neuromuscular blockade to promote a rhythmic breathing pattern to help combat CTBD. Additional efforts to overcome CTBD through employing best practices both pre- and peri-procedure are of interest.

CBCT allows for real time visualization of the lesion and biopsy tool interaction, with the potential for positively impacting diagnostic yield. Studies evaluating the diagnostic yield of ENB guided biopsies in large multicenter registries [24], prospective trials [25], and meta-analyses [13,14,26,27] have shown varying results from 38.5% [24] to 73% [25] to 70–77% [13,14,26,27]. The use of CBCT as the sole secondary confirmation tool [28,29] or in addition to other confirmatory tools [20,30,31] may improve diagnostic yield up to 90% compared to guided bronchoscopy alone, although these results are from studies involving fewer lesions (range of nodules 20–59). This impact stems partially from the information gained from CBCT when visualizing the catheter and biopsy tool in relation to the nodule. Of the 17 nodules biopsied in our cohort, only 24% (four in 17) showed a concentric REBUS pattern prior to CBCT (Table 2), which would suggest a poor diagnostic yield [14,27,32,33]. However, the real-time CBCT feedback allowed for guided, minor adjustments to help improve diagnostic yield to 76% (13 in 17) (Table 3), which would be challenging otherwise given the lack of real-time knowledge that is an inherent limitation of ENB. Thus, the addition of CBCT likely positively impacted our diagnostic yield, making it comparable to other prospective ENB guided studies [25] and meta-analyses [26]. Although most of the nodules were small (59% (10/17) < 2 cm in all dimensions) and without a bronchus sign (65% (11/17)), diagnostic yield for these historically more challenging lesions was acceptable [14,34], which again may be a reflection of the additional use of CBCT to make intraprocedural adjustments (Table 4). Even with CBCT, our overall diagnostic yield remained less than the ~90% rate typically associated with TTNA [11]. Thus, efforts to improve the diagnostic yield of bronchoscopic biopsies are essential.

Novel bronchoscopy modalities are now commercially available with data emerging regarding their diagnostic yields. The Illumisite^TM^ system (Medtronic, Minneapolis, MN, USA) combines fluoroscopic navigation with digital tomosynthesis to correct for CTBD and utilizes continuous EWC catheter localization to provide real-time information on biopsy tool interaction with the target nodule. Initial diagnostic yields are greater than 80% [18,19,35]. Although limited, results for patients undergoing the procedure with moderate sedation rather than general anesthesia show a decreased diagnostic yield [35], suggesting again the importance of not only knowing the lesion location in real time, but also controlling the respiratory cycle. Robotic-assisted bronchoscopy (RAB) platforms seek to improve access to peripheral lung lesions while maintaining catheter stability and shape. The Monarch Platform (Auris Health, Inc., Redwood City, CA, USA) uses an external electromagnetic field generator to localize and track sensors in the robotic catheter and matches these signals with the virtual map that was created by the preprocedural chest CT scan [36]. Early results for ENB RAB show a navigation success rate of 88.6% and diagnostic yield of 69.1% [37]. In contrast, the Ion Endoluminal Robotic Bronchoscopy System (Intuitive, Sunnyvale, CA, USA) uses shape-sensing RAB (ssRAB) technology, which involves a specialized fiber embedded along the robotic catheter [38]. This technology provides real-time information on the shape and location of the target lesions, which can be verified by the chest CT scan derived virtual map. A recent observational study showed that the navigational success rate using ssRAB technology was 98.7% and the diagnostic yield was 81.7% [39]. The combination of ssRAB with CBCT has increased the diagnostic yield to 83% [29]. Determining the impact of these novel technologies with or without adjunct modalities such as CBCT for additional confirmation will require further studies, including potentially head-to-head studies and comparisons to TTNA.

There are several limitations to our study, including its design as a single-center, observational, uncontrolled study with a small sample size. However, our demographics and nodule characteristics were comparable to other studies [25,26]. No patients were excluded during the study period and no run-in cases were removed from the final analysis. Our study presents a real-life, unbiased clinical evaluation that further adds to growing literature for CBCT guided ENB with acceptable diagnostic results and limited safety concerns. Efforts to improve diagnostic accuracy are needed and may focus on improving preprocedure airway algorithms for creation and integration of the virtual bronchoscopy portion of the procedure, standardized anesthesia practices to reduce atelectasis and CTBD, improved biopsy tools to allow for additional articulation and manipulation, and a better understanding of the benefits of advanced imaging modalities like CBCT in the procedural workflow. We also acknowledge the potential issues relating to access to CBCT equipped rooms, which are costly and require additional training for ancillary staff to support their use. However, these issues may be mitigated by resource sharing amongst numerous stakeholders (e.g., interventional radiology, interventional cardiology, and vascular surgery). Similarly, the use of CBCT exposes patients to additional radiation compared to non-CBCT guided bronchoscopy approaches. However, our average number of CBCT spins (3.5+/−1.5) appears to be comparable to other studies performing CBCT guided biopsies alone [40]. Ultimately, the potential for increased radiation exposure will require additional discussion between the patient and the provider to optimize procedural success, minimize risk, and maximize patient satisfaction with the entire experience.

## 5. Conclusions

Accurately obtaining diagnostic tissue from bronchoscopic lung nodule biopsies remains challenging. Novel technologies have the potential to improve this process by overcoming limitations from CTBD through providing real time information regarding lesion location and biopsy tool–lesion interaction. Our initial results combining ENB with CBCT for secondary confirmation support a comparable diagnostic accuracy and acceptable safety profile for this approach compared to other mature methods. Further efforts to optimize this process and to ensure a high diagnostic yield with minimal complications are essential. 

## Figures and Tables

**Table 1:** Patient characteristics.

Characteristic	Mean (Range)
Age	71 (62–84)
**Race/Ethnicity**	**N (%)**
White	12 (86)
Black	2 (14)
**Sex**	**N (%)**
Female	6 (43)
Male	8 (57)
**Smoking Status**	**N (%)**
Never	5 (36)
Former or Current	9 (64)
**History of Cancer**	**N (%)**
Previous cancer	6 (43)

Data are expressed as mean (range) or number (%).

**Table 2:** Nodule Characteristics.

Size	(Mean ± SD) [Range], mm
Axial diameter	21.7 ± 14.9 [9–62]
Coronal diameter	13.2 ± 4.8 [6–46]
	**N (%)**
<2 cm in all dimensions	10 (59)
>2 cm in any dimension	7 (41)
**Type**	**N (%)**
Solid	14 (82)
Mixed	1 (6)
Ground glass	2 (12)
**Location**	**N (%)**
Left Lower Lobe	3 (18)
Left Upper Lobe	3 (18)
Right Lower Lobe	1 (6)
Right Middle Lobe	2 (12)
Right Upper Lobe	8 (47)
**Bronchus Sign**	**N (%)**
Present	6 (35)
Absent	11 (65)
**REBUS**	**N (%)**
Concentric	4 (24)
Eccentric	13 (76)

Data are expressed as mean ± standard deviation, range, or number (percent). REBUS = radial endobronchial ultrasound, SD = standard deviation.

**Table 3:** Biopsy Results.

Biopsy Result	Pathology	N (%)
**Malignancy**	Squamous Cell Carcinoma	4 (23)
	Metastatic disease *	3 (18)
	Adenocarcinoma	2 (12)
	Small Cell Carcinoma	1 (6)
**Inflammation**	Chronic Inflammation	1 (6)
**Infectious**	Non-tuberculous mycobacteria	2 (12)
**Non-diagnostic**		4 (23)

Data are expressed as number (%). * includes bladder cancer (1), endometrial cancer (2).

**Table 4:** Nodule Level Data Results.

Nodule	Size (mm)	Presence of Bronchus Sign	REBUS View *	Biopsy Tool-Lesion Relationship **	Diagnosis
1	34	Positive	Concentric	Within	Chronic Inflammation
2	22	Negative	Concentric	Within	Squamous Cell Lung Carcinoma
3	9.7	Negative	Eccentric	Adjacent	Mycobacterium avium Complex
4	12.4	Negative	Eccentric	Adjacent	Mycobacterium avium Complex
5	24	Negative	Concentric	Within	Lung Adenocarcinoma
6	29	Positive	Concentric	Within	Lung Adenocarcinoma
7	15	Negative	Eccentric	Within	Endometrial Adenocarcinoma
8	11	Negative	Eccentric	Adjacent	Endometrial Adenocarcinoma
9	18	Positive	Eccentric	None	Squamous Cell Lung Carcinoma
10	50	Positive	Eccentric	Adjacent	Squamous Cell Lung Carcinoma
11	23	Negative	Eccentric	Adjacent	Small Cell Carcinoma
12	62	Positive	Eccentric	Within	Non-diagnostic
13	13	Positive	Eccentric	Within	Non-diagnostic
14	15	Negative	None	None	Urothelial Carcinoma
15	11	Negative	None	Adjacent	Non-diagnostic
16	11	Negative	None	None	Non-diagnostic
17	9	Negative	Eccentric	Adjacent	Squamous Cell Lung Carcinoma

REBUS = radial endobronchial ultrasound. * view on radial endobronchial ultrasound was evaluated after electromagnetic navigation was performed, but prior to initial placement of biopsy tool and initial cone beam computed tomography spin. ** biopsy tool-lesion relationship refers to findings after initial cone beam computed tomography evaluation and was categorized as within (biopsy tool visualized inside of lesion), adjacent (biopsy tool visualized next to lesion, but not inside) or absent (no evidence of biopsy tool inside or next to lesion).

## Abbreviations

CBCT	cone beam CT
CT	computed tomography
CTBD	CT scan to body divergence
ENB	electromagnetic navigation guided biopsy
EWC	extended working channel
LG	locatable guide
RAB	robotic-assisted bronchoscopy
REBUS	radial endobronchial ultrasound
ssRAB	shape-sensing robotic-assisted bronchoscopy
TTNA	transthoracic needle aspiration
